# Fabrication of a flexible transparent superomniphobic polydimethylsiloxane surface with a micropillar array†

**DOI:** 10.1039/c9ra04706a

**Published:** 2019-08-21

**Authors:** Shengyang Pan, Min Chen, Limin Wu

**Affiliations:** Department of Materials Science, Advanced Coatings Research Center of Ministry of Education, Fudan University Shanghai 200433 China Lmw@fudan.edu.cn

## Abstract

Although superomniphobic surfaces have attracted extensive interest owing to many important applications, successful fabrication of such surfaces still remains a critical challenge. Herein, we present a flexible transparent superomniphobic polydimethylsiloxane (PDMS) surface with a micropillar array using Si nanowires as the mould. The as-obtained PDMS not only exhibits excellent liquid-repellent performance with a static contact angle of over 150° and sliding angle of less than 6° against a wide range of liquids, but also maintains the super-repellency even under acid/base corrosion, mechanical damage, and unidirectional stretching.

## Abstract 2

A flexible transparent superomniphobic polydimethylsiloxane (PDMS) surface with a micropillar array has been fabricated using Si nanowires as the mould.

## Graphical abstract

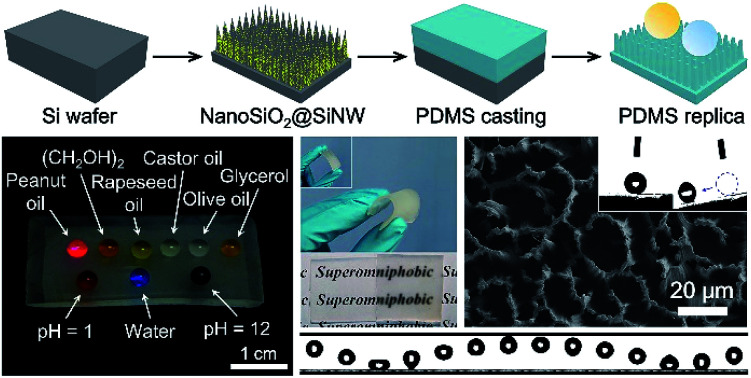

## 1. Introduction

Superomniphobic surfaces that can repel a wide range of liquids with high contact angle and low sliding angle have received increasing attention as they have versatile applications, including antibiofouling,^1,2^ liquid transportation,^3,4^ drag resistance reduction^5,6^ and self-cleaning surfaces,^7–10^*etc.* Building such surfaces usually requires the combination of micro-nanostructured hierarchical surfaces and the subsequent treatment of low surface energy chemicals^11^ The chemical treatment can be implemented by perfluorocarbon materials onto structured surfaces, including vapor deposition^12–15^ and dip-coating.^7,8,16–18^ To make hierarchical micro/nano structures, methods such as stacking micro and nanospheres,^9,16^ texturing fibers,^17^ and spray coating,^7,12,18,19^ have been commonly used while fabrication of such surfaces with special surface topography such as nanopatterned micropillars, usually involves photolithography or reactive ion-etching using sophisticated tools with complex preparation process.^13–15,19–24^

Recently, the unique structure observed on the skin of springtails has been reported to have extraordinary liquid-repellent characteristics.^25^ It is believed that the so-called doubly-re-entrant structures attribute to the super-repellency. Inspired by the surface structure of springtail skin, we have successfully fabricated bio-inspired superomniphobic surface mimicking this doubly-re-entrant structure,^9,10^ through simply heat-treating silica colloidal templates combined with the fabrication of polymer inverse opals. In addition, several works on transparent and flexible PDMS-based superomniphobic surfaces have been reported. For example, Dufour *et al.*^15^ prepared a transparent flexible mushroom-like PDMS surface by moulding process, by two-step photolithography-etching procedure of bilayer photoresists. Golovin *et al.*^19^ also used photolithography to fabricate silicon micro holes array as mould and replicated the flexible transparent PDMS micropillars array that was spray-coated with fluorine-functionalized polyhedral oligomeric silsesquioxane to generate roughness and lower the surface energy. Similarly, Kang *et al.*^14^ and Papadopoulos *et al.*^21^ also fabricated superhydrophobic PDMS surfaces with photolithography with the photoresists. Biria *et al.*^26^ fabricated a series of superhydrophobic microporous substrates with the surface morphologies of interconnected corrugation by solvent removal in the photopolymer–solvent mixture using a patterned photomask. The as-obtained polymer corrugation surface was then modified with polytetrafluoroethylene nanoparticles to be superhydrophobic.

Here, we further report the first flexible transparent superomniphobic polydimethylsiloxane (PDMS) micropillar-based surface that can repel various liquids, including low surface tension oils by lithography using Si nanowires (SiNW) as templates. We first used wet chemistry etching to fabricate a series of SiNW moulds with different void sizes and different heights. These SiNW moulds were then decorated by nanosilica to enhance the roughness of surface microstructure. By moulding–demoulding procedure, the PDMS elastomer replica was obtained and subsequently surface-modified with low-surface-energy trichloro (1*H*,1*H*,2*H*,2*H*-perfluorooctyl) silane (PFOTS). The optimum PDMS micropillar structure not only exhibits excellent liquid-repellent properties with static contact angle (≥150°) and sliding angle (≤6°) for a wide range of liquids, but also maintains the superomniphobicity under acid/base corrosion, mechanical damage, and unidirectional stretch. Compared with other works using photolithography to fabricate the template, our work simplifies the preparation process without sophisticated instruments for photolithography or reactive ion etching.

## 2. Experimental

### 2.1 Materials

Polydimethylsiloxane precursor and its curing agent (PDMS, Sylgard 184) were supplied by Dow Corning Corporation (USA). SiO_2_ sol (diameter ∼20 nm) was purchased from Jiangsu Guolian Technology Co., Ltd. H_2_O_2_ solution (AR, 30 wt%), hydrofluoric acid (HF, AR, 40 wt%), hydrochloride acid (HCl, AR, 36–38%), toluene (AR, 99.5%), acetone (AR, 99.5%) and glycerol (AR, 99%) were purchased from Sinopharm Chemical Reagent Co., Ltd. Poly(diallyldimethylammonium chloride) solution (PDADMAC, 20 wt% in water, *M*_w_ = 100 000–200 000), sodium hydroxide (NaOH, AR, 96%), ethylene glycol (AR, 98%), castor oil (CP), olive oil (CP), dyes of water soluble erioglaucine disodium salt (AR, blue-colored) and oil soluble tartrazine (85%, yellow-colored) and oil red o (75%, red-colored) were purchased from Aladdin Chemical Reagent Co., Ltd. Trichloro(1*H*,1*H*,2*H*,2*H*-perfluorooctyl)silane (CF_3_(CF_2_)_5_CH_2_CH_2_SiCl_3_, PFOTS, 97%) was purchased from Sigma Aldrich. Rapeseed oil and peanut oil were purchased from Walmart. All the reagents mentioned were used as received. Deionized water (18.2 mS conductivity) was used in this work.

### 2.2 Fabrication of SiNW by metal-assisted chemical etching

A pre-cleaned boron-doped p-type Si (100) wafer (2 × 2 cm^2^) was immersed in 1% HF for 1 min to remove the oxide layer, then washed with H_2_O, and dried at room temperature. The Si wafer was immediately placed into 30 mL of 10% HF solution mixed with 25.5 mg of AgNO_3_ (5 mmol L^−1^) for 1 min, 2 min, 3 min, 5 min and 10 min, respectively, to load the Ag catalyst. The Ag-coated Si wafer was washed with water, dried at room temperature, and immediately immersed in the mixture of 40 mL HF solution containing 0.542 g H_2_O_2_ (0.4 mmol L^−1^) for 6 h etching. The obtained SiNW was rinsed and kept in water for further use.

### 2.3 Nanosilica decoration of SiNW wafer and its fluorination

The obtained SiNW was first put in 0.1 wt% of cationic PDADMAC solution for 15 min to adhere the cationic polymer. After rinsed once with water, the SiNW wafer was immediately placed in 1 wt% SiO_2_ dispersion for 5 min for nanosilica decoration. The nanosilica decorated SiNW wafer was washed with water and dried at room temperature.

To release the cured PDMS off the template, the nanosilica decorated SiNW wafer was treated with PFOTS, to form an anti-adhesive layer by placed together with 200 μL of PFOTS in a vacuum desiccator *via* vapor deposition method. The desiccator was pumped to vacuum for 15 min and placed at room temperature for 24 h for fluorination. The fluorinated wafer was then baked in an oven at 120 °C for 1 h to remove the excessive PFOTS.

### 2.4 Fabrication of PDMS flexible micropillar replica surface

PDMS elastomer and its crosslinker were added into a beaker and diluted by toluene at the weight ratio of 10 : 1 : 2.5 for PDMS : crosslinker : toluene, and then evacuated for 30 min to remove the inner air bubbles. This solution was poured gently onto the nanosilica decorated SiNW template for moulding, and then carefully evacuated at ambient temperature for 30 min to let the PDMS solution be properly penetrated into the voids of SiNW. After heated to 80 °C for 3 h for curing PDMS, the micropillar patterned PDMS transparent replica was carefully detached from the SiNW wafer by hand. To achieve superamphiphobicity, the obtained PDMS replica was further surface-fluorinated after oxygen plasma treatment.

### 2.5 Structure characterization

The morphologies of nanosilica decorated SiNW template and the PDMS micropillar replica were observed by Field-Emission Scan Electron Microscope (Zeiss, Ultra 55, Germany). Energy-dispersive X-ray spectroscopy EDX (AZtec X-Max Extreme, Oxford instrument, UK) attached to the Field-Emission Scan Electron Microscope (Zeiss, Ultra 55, Germany) was used to analysis fluorine element of the PDMS replica.

### 2.6 Liquid repellency on the PDMS micropillar surface

To identify the liquid repellency of the fluorinated PDMS micropillar surface, water, ethylene glycol, glycerol, rapeseed oil, olive oil, castor oil and peanut oil were selected as test liquids. Contact angle (CA) and contact angle hysteresis were determined with a contact angle analyzer (TBU 95, DataPhysics Instruments GmbH, Germany). 3 μL of liquid droplet was dropped onto the PDMS micropillar surface to confirm its liquid repellency. The mean value of CAs from three different sites on the same specimen was adopted.

### 2.7 Stability measurements

For the effect of pH, the samples were immersed in acidic and alkaline solutions (pH: from 1 to 13) for 24 h. To demonstrate the PDMS replica with nanopatterned micropillar has good mechanical strength, dumbbell-shaped test pieces (0.5–1 mm thick and 4.3 mm wide) were used for stress *versus* extension ratio using an Instron machine (model 5966, USA) with the uniaxial extension setup at 50 mm min^−1^ until fracture.

The mechanical property of the patterned PDMS replica was further characterized by Sand Abrasion Test referring to an American Standard (ASTM Designation: D 968-93). Sandpaper scratching test was introduced by dragging the PDMS sample loaded by 100 g weight on the top to scratch on a piece of sandpaper (1500 mesh) for 10 cycles. Compressive test was performed by placing a 300 g weight on the micropillar surface, with a contact area of 4 cm^2^. Moreover, a Chinese coin (1 Jiao) was attached to one side of the weight to provide a more concentrated and greater stress on the surface.^27^ The micropillar morphologies were observed after the test and the super-repellency was identified by water and glycerol spreading over the surface after the application of compression stress.

### 2.8 Optical measurement

UV-vis spectroscopy measurements were made on a Lambda750 UV/Vis spectrophotometer (PerkinElmer, US). The sample (2 × 2 cm^2^) was scanned in the wavelength range of 400–800 nm.

## 3. Results and discussion

### 3.1 Fabrication of SiNW and PDMS replica

**Scheme 1:**
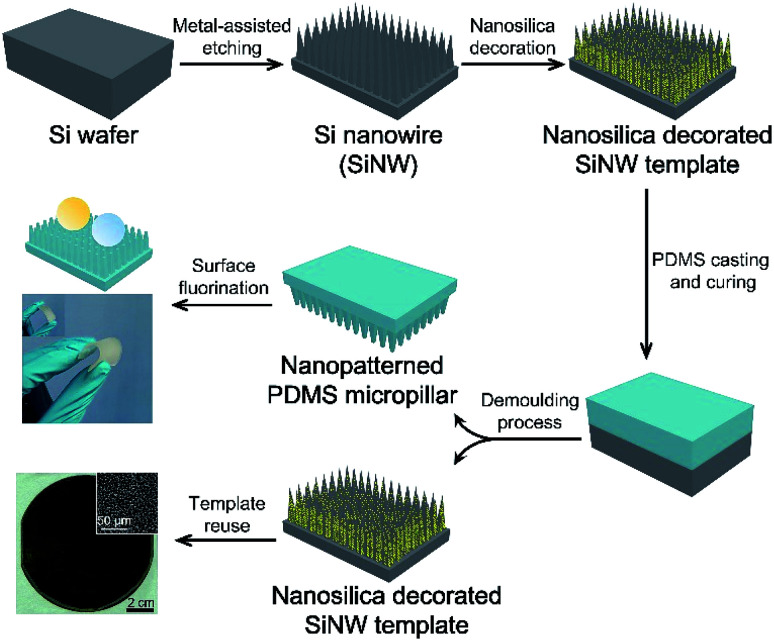
Fabrication of the flexible transparent superomniphobic PDMS micropillar-based surface using nanosilica decorated SiNW as template.

**Fig. 1:**
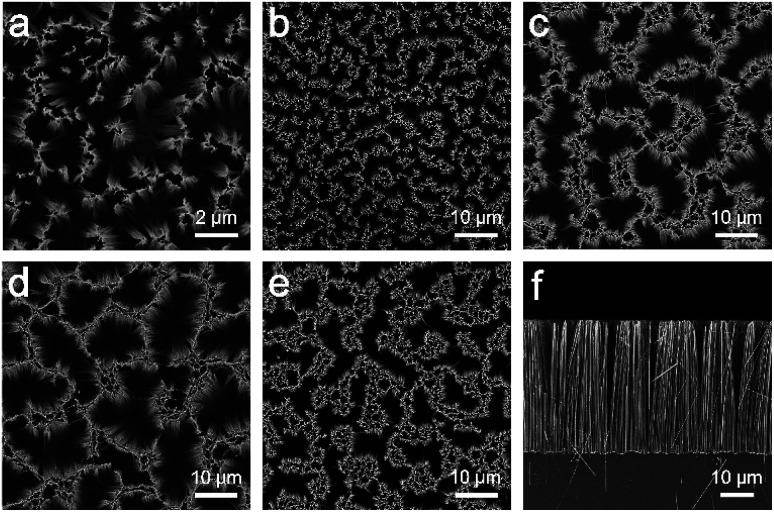
(a)–(e) FESEM top-view images of the nanosilica-decorated SiNW template with different plating time and spacings; (a) 1, (b) 2, (c) 3, (d) 5, (e) 10 min. (f) FESEM cross-sectional image of the SiNW template from 5 min-plating.

The SiNW structures and PDMS replica are fabricated as shown in Scheme 1. In brief, Ag metal particles were firstly deposited on the clean Si wafer in AgNO_3_/HF solution, and the wafer is subsequently etched in H_2_O_2_/HF to obtain SiNW structure.^28^ The morphologies of the resulting structure are influenced by the distance between metal catalyst particles because the Si under Ag particle catalyst is etched much faster than Si without metal coverage.^29^ In this case, SiNW wafers with various spacings can be fabricated by adjusting the plating time of Ag catalyst. As the plating time increases from 1 min, 2 min, and 3 min to 5 min, the spacing between nanowires increases with increasing cone-shaped pore size from 1 μm to 20 μm (see Fig. 1a–e). When the plating time is extended to 10 min, the SiNW spacing decreases owing to the “overlapping” behavior of the high particle density of Ag catalysts on the wafer surface while etching.^30^ The degree of the etching is indicated by the depth of the cone-shaped pore. The optimum 40 μm deep pore is obtained (see cross-sectional image in Fig. 1f), to balance the facility of demoulding process of PDMS replica and the structural design for achieving superomniphobicity.

**Fig. 2:**
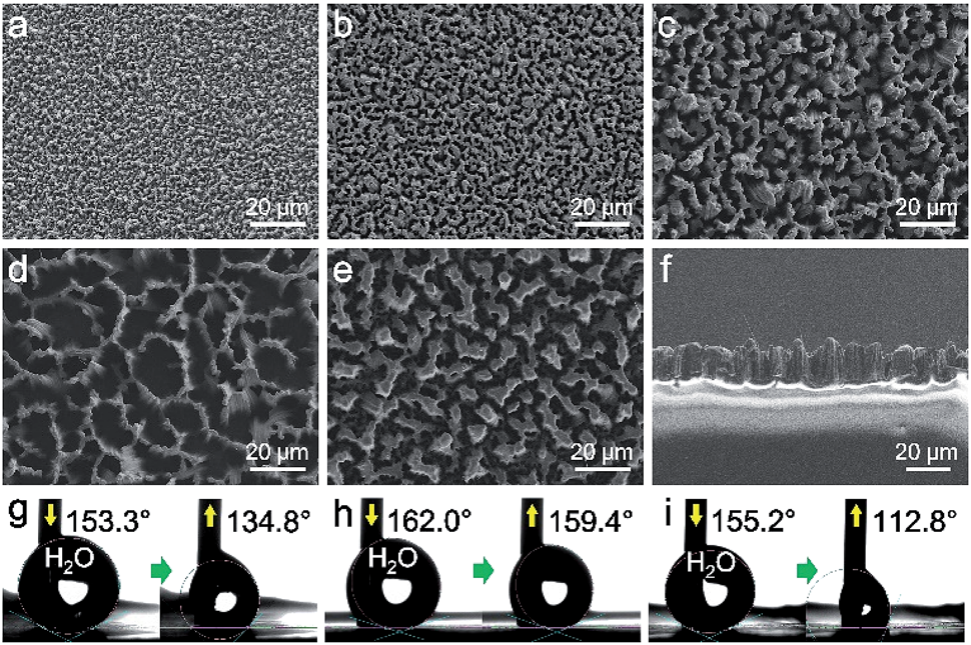
(a)–(e) FESEM top-view images of surface-fluorinated PDMS replica after demoulding from SiNW template; (a) replica from 1 min-plating SiNW, (b) replica from 2 min-plating SiNW, (c) replica from 3 min-plating SiNW, (d) replica from 5 min-plating SiNW, (e) replica from 10 min-plating SiNW. (f) FESEM cross-sectional image of replica from 5 min-plating SiNW. (g)–(i) Water CAs on the replicas from 3 min-, 5 min- and 10 min-plating SiNW, respectively.

Due to different spacings in SiNW mould, the demoulded PDMS replicas present different morphologies. As shown in FESEM images (Fig. 2a–e), all the PDMS replicas present comparable dimensions to their SiNW moulds (see cross-sectional image in Fig. 2f). The SiNW template could be reused for the replication of PDMS. As shown in Fig. S1,† both the SiNW template and the demoulded PDMS replica present similar spacings, pore sizes and heights compared to the former ones.

### 3.2 Liquid repellency of the PDMS replica

**Fig. 3:**
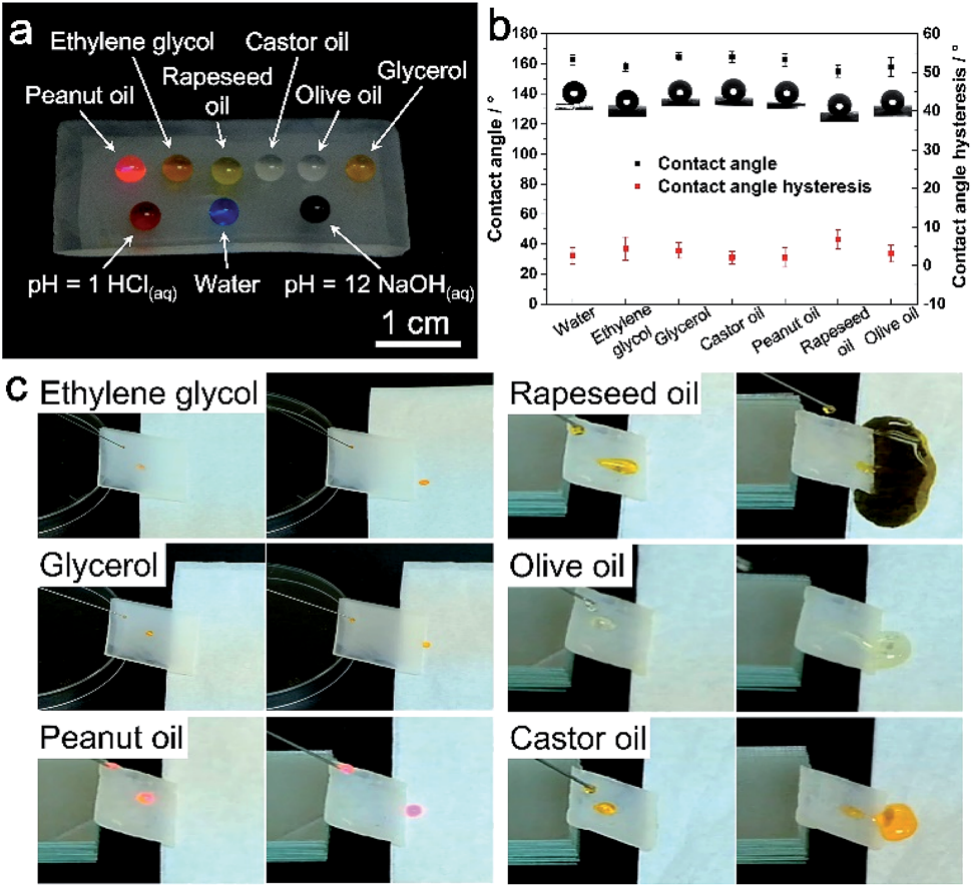
(a) Digital photographs of liquid sitting on the PDMS replica from 5 min-plating time SiNW. (b) CAs and CAHs of the liquid droplet on this PDMS replica surface. (c) Photographs of oil droplets rolling down the slope surface of replica.

The PDMS replicas were then surface-modified with PFOTS to achieve oil repellency. EDS mapping confirmed element F has been coated onto the replica surface after surface modification (Fig. S2†). For the low surface tension oil droplets, the deposited PFOTS layer on the PDMS surface not only lowers the CAH, but also reduces PDMS swelling when exposed to low surface tension liquids.^15^ The static contact angles (CA) and the contact angle hysteresis (CAH) of water and various oils on the PDMS replica surface were recorded and calculated, respectively. As shown in Table 1, the PDMS replica from 5 min-plating SiNW exhibits the best superomniphobic ability. All the test liquid droplets dyed in colors are sitting on this replica surface in nearly perfect spherical shapes (Fig. 3a) and all their CAs are greater than 150° with the CAH of less than 6° (Fig. 3b), indicating excellent superomniphobicity.

**Table 1:** CAs and CAH of various liquids on PDMS-based surface

CA (CAH) of replicas from different plating time SiNW/°	Water	(CH_2_OH)_2_	Glycerol	Castor oil	Peanut oil	Rapeseed oil	Olive oil
Flat PDMS^a^	119.6	117.0	117.5	111.4	112.8	104.1	94.2
1 min-plating SiNW	137.7	133.3	136.2	118.2	117.5	117.2	125.9
2 min-plating SiNW	149.4	138.2	136.5	130.1	123.9	122.5	129.7
3 min-plating SiNW	153.6	154.1	159.8	148.3	152.3	143.2	146.3
	(18.5)						
5 min-plating SiNW	162.6	158.1	164.7	164.5	164.5	155.0	158.0
	(2.6)	(4.4)	(3.9)	(2.1)	(2.1)	(6.8)	(3.1)
10 min-plating SiNW	150.6	140.5	143.2	140.2	138.6	141.6	139.2
	(42.4)						

a The contact angle for flat PDMS modified by surface-fluorination represents the intrinsic contact angle *θ*_Y_ of surface-fluorinated PDMS material.

To further investigate the impact of micropillars' height on the superomniphobicity of PDMS surfaces, various SiNW templates with different etch time were prepared. As shown in Fig. S3a–d,† the etch time of Si wafer increases from 30 min to 6 h, resulting in the increase of SiNWs height, from 10 μm to 70 μm, respectively. Si wafer etched for longer time (9 h) was also investigated, the cross-sectional SEM image shown in Fig. S3e† and its insets indicate the height of the SiNWs is 150 μm, but no applicable spacings and pores are formed on the surface after long time etch, causing little roughness of “tiny raise” on the replicated PDMS surface (Fig. S3j†). According to Fig. S3f–i,† the height of the replicated pillars increases from 2 μm to 20 μm, respectively. The CA and CAH of liquids on PDMS surfaces with different pillar height were also recorded. As shown in Table S1,† PDMS pillar with the height of H_1_ ∼2 μm and H_2_ ∼5 μm present poor superhydrophobicity with water CA less than 150° and water CAH greater than 30°. For pillars with height of H_3_ ∼10 μm, the PDMS surface presents water CA of 153.8° and oil CAs around 140°, but water CAH of as high as 16.2°, indicating poor non-stick ability. The aforementioned PDMS replica from 5 min-plating SiNW presents the pillar height of 20 μm (Fig. S3i†), which has the best liquid repellent performance according to Tables 1 and S1.†

The PDMS replica from 5 min-plating SiNW was further used for investigation of the liquid sliding and non-stick ability. As shown in Fig. 3c and ESI Video Movie S3,† all the liquid droplets can roll down from the PDMS replica surface without any residues.

In contrast, other PDMS replicas seem not to have as nice superomniphobicity as the replica from 5 min-plating SiNW: The replicas from 1 min- and 2 min-plating SiNW have Water CAs less than 150° and the olive oil CAs less than 130°. The replica from 3 min-plating SiNW presents WCA of 153° and the oil CA of around 150°, but CAH of as high as 18°. For the replica from 10 min-plating SiNW, its water CAH even reaches 42° in spite of its water CA of 150°, indicating poor non-stick ability. According to Cassie–Baxter model:

1 cos θ* = *f*_s_ cos *θ*_Y_ − *f*_g_

where θ* is apparent contact angle and measured by contact angle analyzer, *θ*_Y_ is the intrinsic contact angle for flat PDMS surface and listed in Table 1. *f*_s_ is the solid fraction, the proportion of liquid–solid contact area, and *f*_g_ is the gas fraction defined as the proportion of liquid–vapor contact area.

Thus the relationship of solid fraction *f*_s_ and gas fraction *f*_g_ with apparent contact angle θ* could be investigated. As is shown in Fig. 2, the replicas (Fig. 2a–c and e) have less spacings than the replica from 5 min-plating SiNW (Fig. 2d). The increasing spacings enable more air to be trapped in micropillars and generate larger proportion of liquid–air contact area, resulting in the decrease of solid fraction *f*_s_.^30^ According to eqn (1), the apparent contact angle θ* can be greatly increased as *f*_s_ decreases and *f*_g_ increases. In fact, the PDMS replica from 5 min-plating SiNW has the largest spacings (20 μm between micropillars) while other replicas possess only 1 to 6 μm spacings.

It is notable that Cassie–Baxter model could be simplified if liquid–solid and liquid–vapor interfaces are perfectly flat, which is also known as the ideal Cassie state with *f*_s_ + *f*_g_ = 1

2 cos θ* = *f*_s_ (1 + cos *θ*_Y_) − 1

although eqn (2) is only applied to ideal Cassie state, knowing the intrinsic water contact angle *θ*_Y_ on flat PDMS surface and the apparent water contact angle θ*, the solid fraction *f*_s_ calculated for each replica surfaces are 0.514, 0.275, 0.206, 0.090 and 0.254 for the replicas from 1 min-, 2 min-, 3 min-, 5 min- and 10 min-plating SiNW, respectively.

Notably, for the replica from 5 min-plating SiNW, the solid fraction *f*_s_ calculated using other oils by eqn (2) are 0.132, 0.128, 0.057, 0.074, 0.124 and 0.079 using ethylene glycol, glycerol, castor oil, peanut oil, rapeseed oil and olive oil, respectively, which are very close to each other, and very close to the value calculated by water contact angle (0.090), indicating the replica 5 min-plating SiNW is approximate to Cassie state surface.

In addition, the nanosilica decoration on the SiNW is also very critical: without nanosilica decoration, the PDMS replica has a high water CA of 154° but strong water adhesion (Fig. S4, ESI†).

Compared with the previously reported PDMS-based surfaces with super-repellency to liquids,^14,15,19,21^ the PDMS micropillar surface we present here displays excellent superomniphobicity with high static contact angle and low contact angle hysteresis. And the nanostructure on PDMS microplillar in our work can be simply achieved by the imprint of decorated nanosilica on the SiNW mould with accurately controllable spacings.

**Fig. 4:**
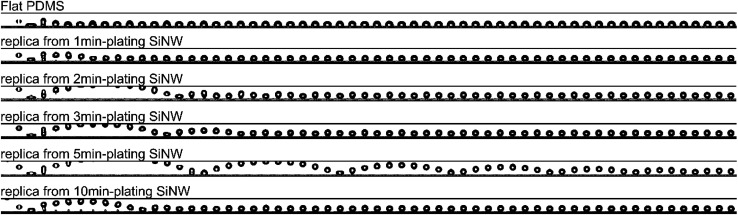
Frame-by-frame picture of 4 μL water droplet bouncing on PDMS flat and replica surfaces from a 1.5 cm height above the surface recorded by a high-speed camera (650 frames per second).

The liquid-repellent performance of these PDMS replicas can be further investigated by their water bouncing behavior. As shown in Fig. 4 and ESI Video Movie S4,† a 4 μL droplet of water was released from a height of 1.5 cm above the PDMS-based surface and a high-speed camera was used to capture the dynamic bouncing behavior of the water droplet on all the PDMS surfaces at room temperature. For flat PDMS surface, as soon as the water droplet touches the surface, it is adhered immediately, without any chance to bounce, which indicates the intrinsic water bouncing behavior of PDMS material. For the replica from 1 min-plating SiNW, the water droplet bounces once at a low height compared to others. At the 7th frame (*t* = 10.78 ms) of its frame-by-frame photographs, water droplet is adhered to the surface and cease to bounce. For the replica from 2 min-plating SiNW, water droplet also bounces one cycle but can achieve higher bouncing height than the replica from 1 min-plating SiNW. It takes the droplet 21.6 ms (at 14th frame) to finish the bounce, indicating the surface is more repellent to water droplet. As to the replica from 3 min-plating SiNW, water droplet can accomplish 2 bounce cycles and the time of first cycle is 20.0 ms which is close to the one of the replica from 2 min-plating SiNW. The replica from 5 min-plating SiNW enables the water droplet to bounce 6 cycles and presents the largest bouncing height of the first cycle (*t* = 24.6 ms, at 16th frame). For the replica from 10 min-plating SiNW, there is only one bouncing with the time of the bounce cycle is 16.9 ms and the part of the droplet is adhered to the surface after first impact. All these results are consistent with liquid contact angle and its hysteresis in Table 1.

The number of bouncing cycles of water droplet on a superhydrophobic surface depends upon the surface microstructure, surface tension and static contact angle.^31–37^ The replica from 1 min-plating SiNW has the lowest water CA (138°). For other PDMS replicas that have comparable water CA (around 150°) and same PFOTS-fluorinated process, the key factor that influences water bouncing behavior is the surface microstructures, that is, the spacings between nanopatterned micropillars. Water droplet bouncing cycles increase with the increasing spacings, indicating better water-repellent ability.

### 3.3 Mechanical strength and chemical stability

**Fig. 5:**
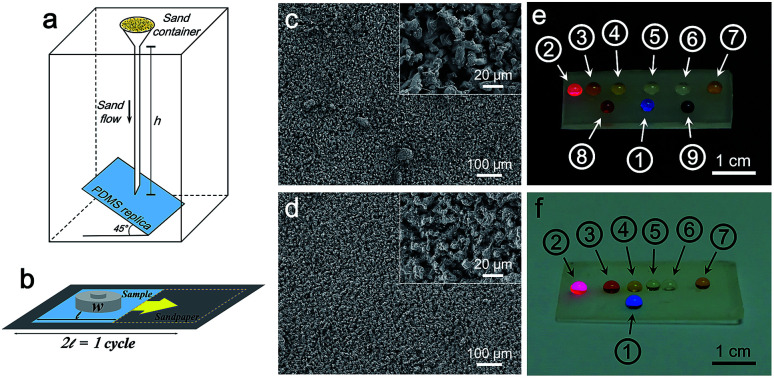
(a) Schematic illustration for sand impact test. (b) Schematic illustration for sandpaper scratch test. (c) and (d) FESEM images of the PDMS replica from 5 min-plating SiNW after sand abrasion test and sandpaper scratch test, respectively. (e) and (f) Digital photographs of liquid droplets (① water, ② peanut oil, ③ ethylene glycol, ④ rapeseed oil, ⑤ castor oil, ⑥ olive oil, ⑦ glycerol, ⑧ pH = 1 HCl_(aq)_, ⑨ pH = 12 NaOH_(aq)_) on the PDMS replica surface after sand abrasion test and sandpaper scratch test, respectively.

To demonstrate the mechanical robustness of PDMS replica, sand abrasion test and sandpaper scratch test were conducted. After the sand abrasion test illustrated in Fig. 5a, the liquid droplets sitting on the PDMS replica from 5 min-plating SiNW still maintain nearly perfect spherical shape (Fig. 5e), because of its robust micropillar structure (Fig. 5c). Even after scratched for 10 cycles (Fig. 5b), the PDMS replica still keeps its good superomniphobicity and micropillar structure (Fig. 5d and f), indicating a robust superomniphobic film.

**Fig. 6:**
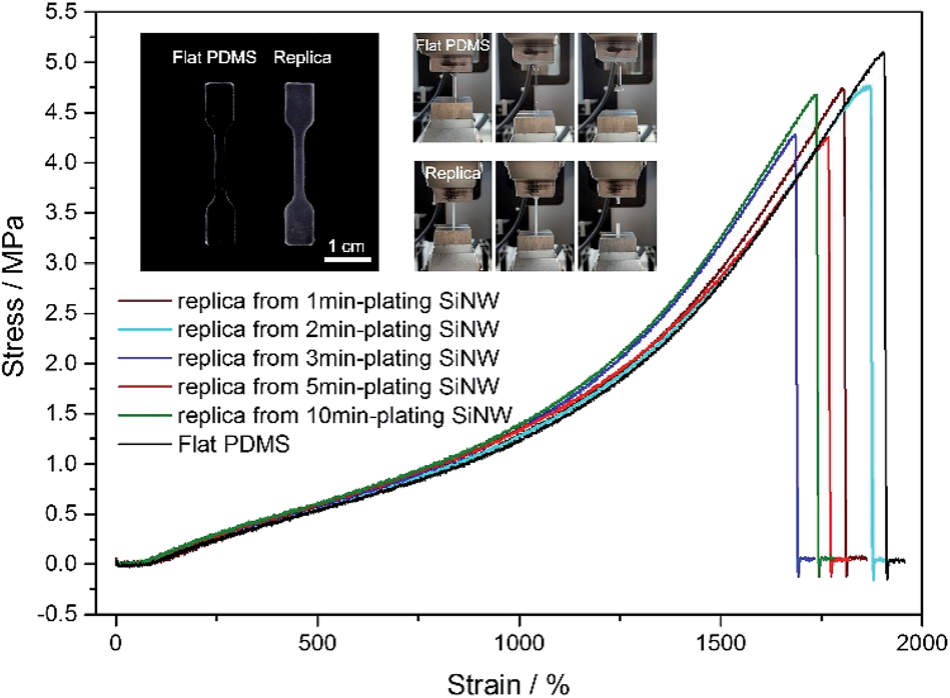
Stress–strain curve of flat PDMS and the PDMS replica samples. Insets: digital photograph of dumbbell-shaped test pieces of flat PDMS and the PDMS replica from 5 min-plating SiNW and digital photographs of samples.

The mechanical strength of PDMS replica was further tested by tensile experiment on an Instron machine. From Fig. 6, flat PDMS sample and PDMS replica samples all presents high fracture stress over 4 MPa and high elasticity with extension ratio over 1700%. The flexible PDMS-based surface is durable, the micropillar can retain the superomniphobicity and structure after the compressive test (see Fig. S5 and ESI Video Movie S5†). No significant damage was observed through the application of stress to the surface.

Although it was reported alkaline solution was able to break down Si–O–Si bond in PDMS network,^38^ in our experiments, after the PDMS replica was immersed in acidic solution (pH = 1, HCl solution) and alkaline solution (pH = 12, NaOH solution) at room temperature for 24 h, respectively, it still keeps its excellent superomniphobicity.

All of these results proves that the PDMS replica we present here has adequate mechanical robustness to sustain long-term liquid-repellency.

### 3.4 Optical transparency

**Fig. 7:**
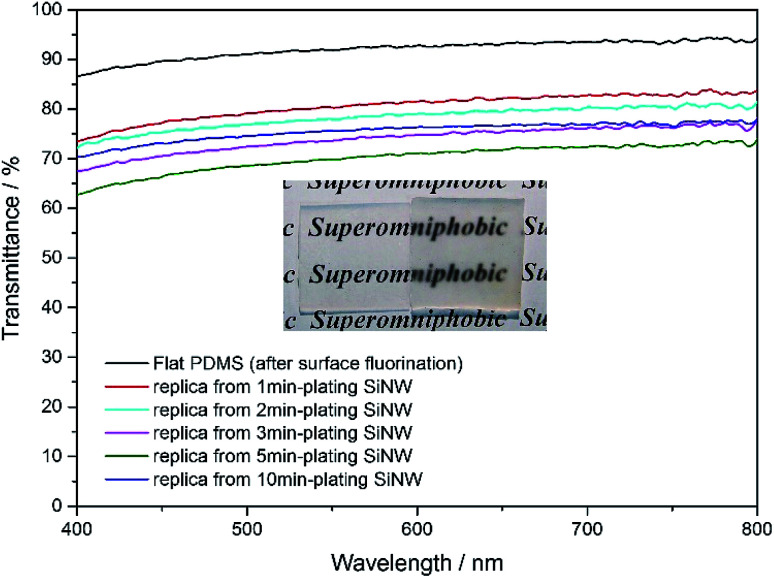
Transmittance spectra of various PDMS samples. Inset: digital photographs of flat PDMS film on the left and replica film from 5 min-plating SiNW on the right.

Compared to surface-fluorination flat PDMS which still has 86–92% of transmittance, the surface-microstructured PDMS replica become semi-transparent, with the visible transmittance of 64–79% as shown in Fig. 7. Obviously, the transparency of the PDMS replica varies with microstructure spacings between micropillars. The larger the spacing between micropillars, the poorer the transparency of the PDMS replica is due to Mie scattering from the rough surface.^39^ Even so, the superomniphobic PDMS replica film still possesses a relatively high transmittance of 72%, the English letters underneath it can still be recognized.

## 4. Conclusions

In summary, we have demonstrated a facile method to fabricate a flexible transparent superomniphobic PDMS film with micropillar array surface that can repel water and various low surface tension liquids by using Si nanowires as mould. The superomniphobic performance of this surface strongly depends on the spacing between micropillars. The as-obtained PDMS micropillar array-based surface with 20 μm spacings not only displays excellent liquid-repellent properties against water and various low surface energy oils, but also sustains a series of chemical and mechanical damage. This semi-transparent, flexible and superomniphobic PDMS-based film may find potentials as solar panels or anti-wetting protective films used for digital screens.

## Conflicts of interest

There are no conflicts to declare.

## Supplementary Material

RA-009-C9RA04706A-s001

RA-009-C9RA04706A-s002

RA-009-C9RA04706A-s003

RA-009-C9RA04706A-s004

RA-009-C9RA04706A-s005

RA-009-C9RA04706A-s006
