# Supplementary material for: Fabrication of a flexible transparent superomniphobic polydimethylsiloxane surface with a micropillar array
Source: RSC Adv. 2019 Aug 21;9(45):26165–71. doi: 10.1039/c9ra04706a (PMC9070391; doi:10.1039/c9ra04706a)
Supplement: RA-009-C9RA04706A-s006 [file RA-009-C9RA04706A-s006.pdf]

Electronic Supplementary Material (ESI) for RSC Advances.
This journal is © The Royal Society of Chemistry 2019

# Support Information

**Fabrication of Flexible Transparent Superomniphobic Polydimethylsiloxane Surface with Micropillar Array**

Shengyang Pan, Min Chen and Limin Wu*

Department of Materials Science and Advanced Coatings Research Center of Ministry of Education, Fudan University, Shanghai 200433, China

* Corresponding author.
E-mail address: lmw@fudan.edu.cn (L. Wu)

**Table S1** CAs and CAH of various liquids on PDMS-based surface with different pillar heights

| CA (CAH) of PDMS replicas with different pillar heights (H) / ° | Water | $(CH_2OH)_2$ | Glycerol | Castor oil | Peanut oil | Rapeseed oil | Olive oil |
|---|---|---|---|---|---|---|---|
| $H_1$ ~ 2 μm: (f) in Fig. S3 | 138.3 (50) | 126.4 | 131.1 | 122.7 | 121.5 | 118.0 | 110.1 |
| $H_2$ ~ 5 μm: (g) in Fig. S3 | 141.5 (30) | 135.3 | 136.8 | 127.2 | 129.3 | 127.4 | 129.7 |
| $H_3$ ~ 10 μm: (h) in Fig. S3 | 153.8 (16.2) | 140.2 | 148.6 | 134.8 | 138.0 | 139.7 | 133.9 |
| $H_4$ ~ 20 μm: (i) in Fig. S3 (replica from 5min-plating SiNW) | 162.6 (2.6) | 158.1 (4.4) | 164.7 (3.9) | 164.5 (2.1) | 162.7 (2.1) | 155.0 (6.8) | 158.0 (3.1) |
| No obvious pillar: (j) in Fig. S3 | 125.9 (>50) | 121.6 | 126.3 | 113.5 | 115.2 | 104.5 | 101.9 |

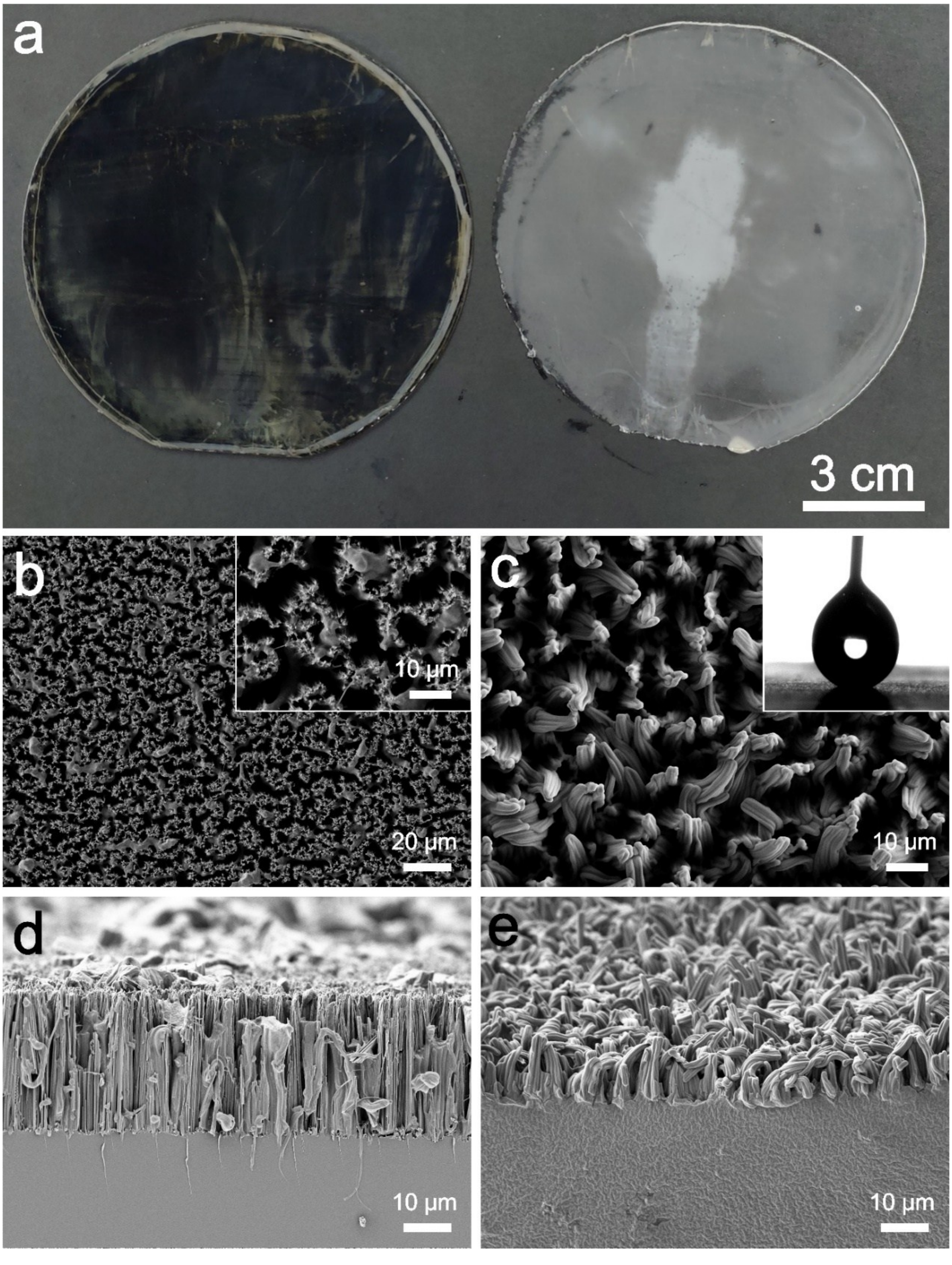


**Fig. S1** (a) Images of reusable SiNW template (left) used for 3rd time and the demoulded PDMS surface (right). (b) and (d) FESEM top-view and cross-sectional images of SiNWs after twice moulding process of PDMS. Insets of (b): magnified image of pores and spacings between SiNWs. (c) and (e) FESEM top-view and cross-sectional images of demoulded PDMS micropillar using reusable SiNW template. Insets of (c): olive oil droplet (4 μL) sitting on the surface of PDMS micropillar with an OCA of 155.2 °

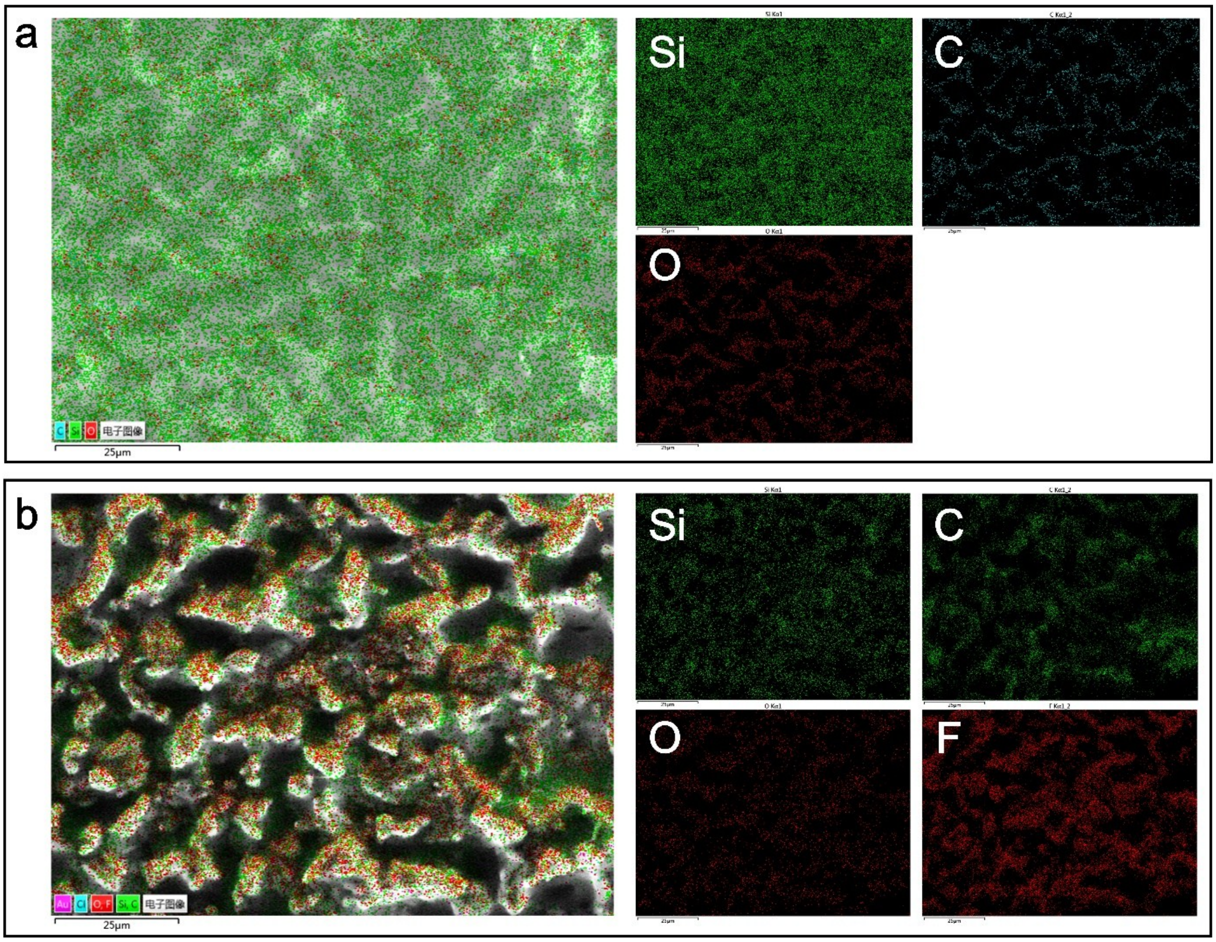


**Fig. S2** EDS mapping of PDMS replica surface before (a) and after (b) surface modification of PFOTS

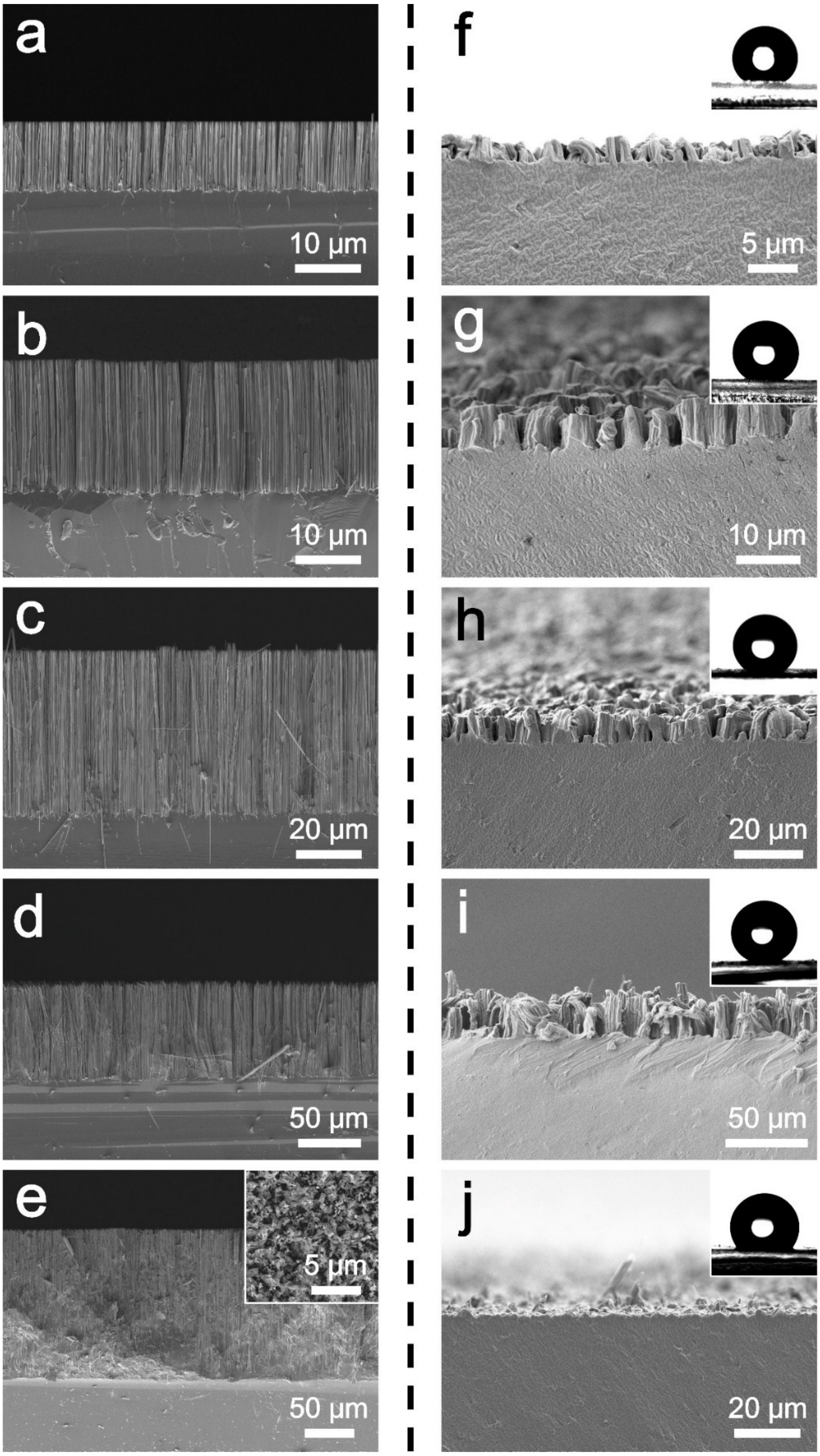


**Fig. S3** (a)-(e) Cross-sectional FESEM images of SiNWs etched with different time: 30 min, 1 h, 3 h, 6 h, 9h, respectively. Insets of (e): surface morphology of Si wafer etched for 9 h. (f)-(j) Cross-sectional FESEM images of PDMS micropillar with different pillar heights after the replication from (a)-(e) respectively. Insets of (f)-(j): water droplet (4 μL) sitting on the PDMS surfaces

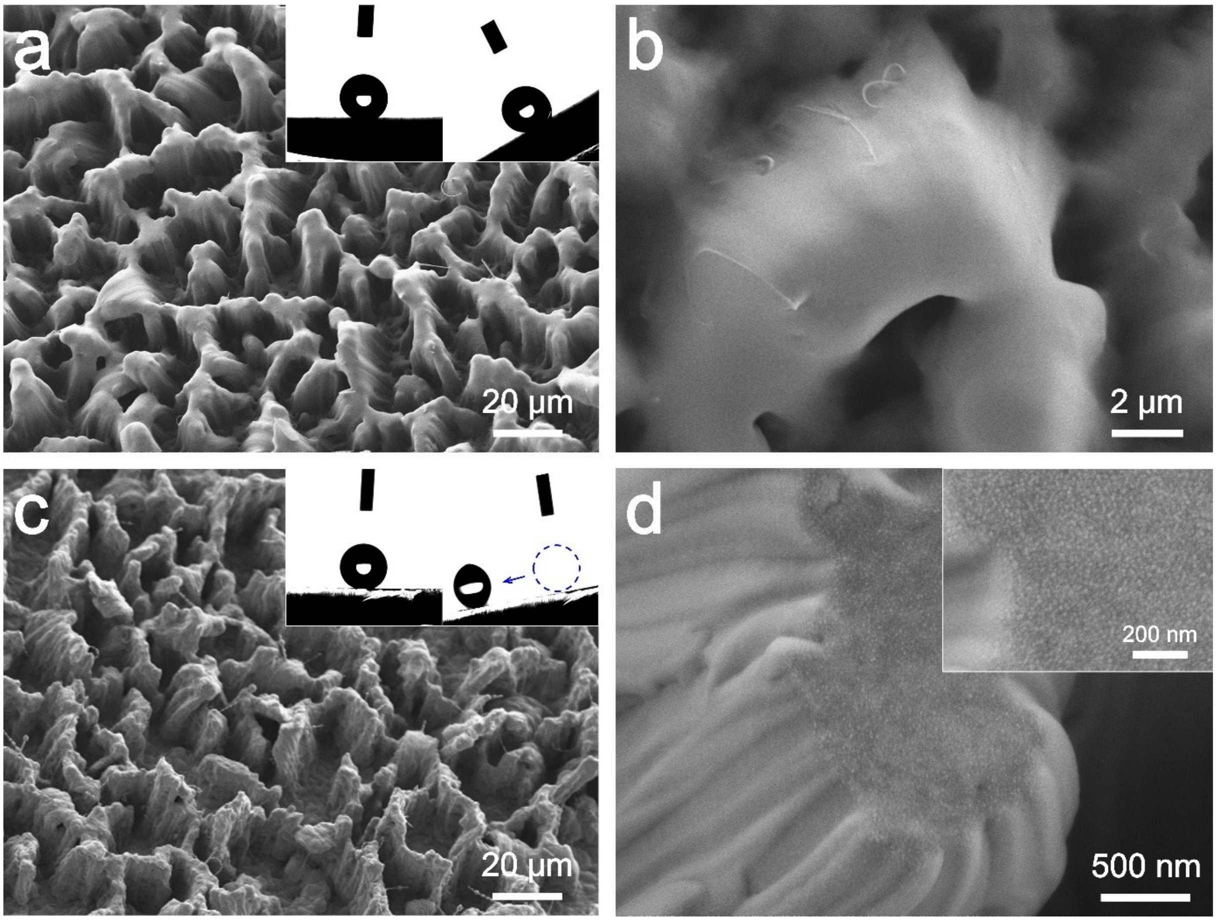


**Fig. S4** (a) and (b) FESEM bird view images of PDMS replica without nanopattern, removed from SiNW template without nanosilica decoration. Insets of (a): water droplet anchored by the surface when tilted to 30 °. (c) and (d) FESEM bird view images of PDMS replica with nanopattern, removed from nanosilica-decorated SiNW template. Insets of (c): water droplet (3 μL) slide when surface tilt angle approaching 12 °. Inset of (d): Magnified nanopattern of PDMS replica

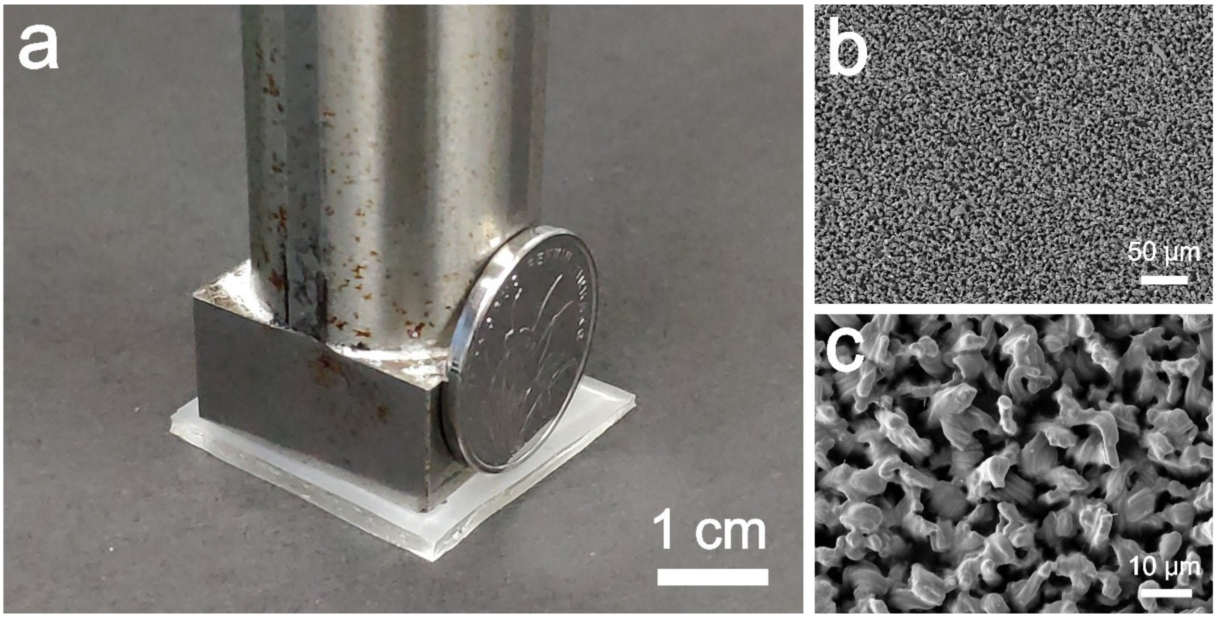


**Fig. S5** (a) Image of a 300 g weight rested on the micropillar array surface. A Chinese coin 1 Jiao was also attached to the weight, for a more localized and greater stress to the surface. (b) and (c) FESEM images of the micropillar morphologies of PDMS surface after compressive test

**Supplementary Videos**

**Movie S1** Dynamic video of contact angle hysteresis measurement of various liquids on PDMS-based surface

**Movie S2** Dynamic video of liquid droplet probe test on PDMS-based surface

**Movie S3** Various liquid droplets rolling down the slope surface of PDMS-based film

**Movie S4** Slow motion video of water droplet bounce on PDMS-based surface captured by high-speed camera

**Movie S5** Compressive test of 300 g weight rested to the PDMS-based surface
